# P95 Assessing the feasibility of implementing AWaRe-based quality indicators into existing antimicrobial use surveillance systems: a data re-use strategy

**DOI:** 10.1093/jacamr/dlaf230.102

**Published:** 2025-12-04

**Authors:** Juliet Sanyu Namugambe, Annie Heath, Hossam Almadhoon, Stephen Campbell, Brian Godman, Michael Sharland, Aislinn Cook

**Affiliations:** City St Georges University, Antibiotic Policy Group, London, UK; City St Georges University, Antibiotic Policy Group, London, UK; City St Georges University, Antibiotic Policy Group, London, UK; University of Manchester, Manchester, UK; School of Pharmacy, Sefako Makgatho Health Sciences University, Ga-Rankuwa, Pretoria, South Africa; City St Georges University, Antibiotic Policy Group, London, UK; School of Pharmacy, Sefako Makgatho Health Sciences University, Ga-Rankuwa, Pretoria, South Africa; Strathclyde Institute of Pharmacy and Biomedical Sciences, Strathclyde University, Glasgow, UK; City St Georges University, Antibiotic Policy Group, London, UK; City St Georges University, Antibiotic Policy Group, London, UK; Nuffield Department of Primary Care Health Sciences, University of Oxford, Oxford, England, UK

## Abstract

**Background:**

AWaRe-based quality indicators (QIs) were developed to measure appropriateness of antibiotic prescribing in line with the WHO AWaRe Antibiotic Book. They comprise 61 inpatients QIs across seven infection groups and surgical prophylaxis and 57 primary care QIs across six infection groups. Antimicrobial surveillance methodologies, such as the WHO Point Prevalence Survey (WHO-PPS) and Global-PPS, which deliver standardized global data on antimicrobial use, may provide suitable platforms for integrating these indicators. Here, we examined the feasibility of integrating AWaRe-QIs into these established surveillance frameworks to enable joint monitoring of antimicrobial consumption and disease-specific prescribing quality data.

**Methods:**

We cross-referenced each QI against data variables routinely collected in WHO-PPS and Global-PPS for inpatient indications and against Global-PPS outpatient protocol for primary care indicators. We mapped each indicator to the diagnosis (inpatient) or presenting symptoms (outpatient) available in each of the PPS protocols. The AWaRe-based QIs assess proportions of prescriptions for empirical therapy of community-acquired infections that were in alignment with the AWaRe Book recommendations. The required data elements to measure the QIs consisted of the diagnosis (type of infection), treatment type (empirical or targeted), indication (community-, hospital acquired, medical or surgical prophylaxis) and antimicrobial data (antibiotic choice, dosage, route, duration, AWaRe category). For outpatient QIs, whether an antibiotic was prescribed and risk classification for some infections were also required.

**Results:**

For inpatient indicators, five of seven diagnoses (sepsis, respiratory tract infections, upper UTIs, intrabdominal infections, skin/soft tissue/osteoarticular infections) were explicitly defined by both WHO-PPS and Global-PPS protocols. Meningitis and diarrhoea were not explicit, but CNS and gastrointestinal infections can be assumed to cover these infections. For inpatient QIs, data on antibiotic choice, total daily dose, route were available; however, data on duration and infection severity were not available in either Global-PPS or WHO-PPS. For surgical prophylaxis, antibiotic choice, dose and duration are collected but not timing of the dose. Therefore, 18/61(30%) inpatient indicators could be measured using WHO-PPS or Global-PPS. For the outpatient QIs, the symptoms listed on the Global-PPS form can be used as a proxy for the diagnosis; diagnosis is only captured for patients prescribed an antibiotic. Within the Global-PPS protocol, antibiotic choice, dosage and prescribed duration were measurable; however, severity and risk criteria required for certain QIs could not be assessed. Therefore, 33/57 (58%) outpatient QIs were measurable using Global-PPS data.

**Conclusions:**

A third of inpatient QIs and over half of outpatient QIs were measurable by the PPS systems. While the infections, antibiotic choice and dosage data were available, key challenges include absence of data on duration of prescription for inpatients and absence of severity/risk classification for inpatients and outpatients. Despite these limitations, a number of AWaRe-QIs were measurable using existing PPS tools demonstrating their practical applicability; they can support antimicrobial stewardship teams to augment the clinical utility of existing data. Feasibility could be enhanced by reasonably expanding PPS data collection elements and developing data analysis platforms for the QIs compatible with PPS data structures.

